# Far-field ultrasonic imaging using hyperlenses

**DOI:** 10.1038/s41598-022-23046-7

**Published:** 2022-10-29

**Authors:** Mohamed Subair Syed Akbar Ali, Prabhu Rajagopal

**Affiliations:** Centre for Nondestructive Evaluation, Department of Mechanical Engineering, Indian Institute of Technology Madras, Chennai, 600036 Tamil Nadu India

**Keywords:** Mechanical engineering, Physics

## Abstract

Hyperlenses for ultrasonic imaging in nondestructive evaluation and non-invasive diagnostics have not been widely discussed, likely due to the lack of understanding on their performance, as well as challenges with reception of the elastic wavefield past fine features. This paper discusses the development and application of a cylindrical hyperlens that can magnify subwavelength features and achieve super-resolution in the far-field. A radially symmetric structure composed of alternating metal and water layers is used to demonstrate the hyperlens. Numerical simulations are used to study the performance of cylindrical hyperlenses with regard to their geometrical parameters in imaging defects separated by a subwavelength distance, gaining insight into their construction for the ultrasonic domain. An elegant extension of the concept of cylindrical hyperlens to flat face hyperlens is also discussed, paving the way for a wider practical implementation of the technique. The paper also presents a novel waveguide-based reception technique that uses a conventional ultrasonic transducer as receiver to capture waves exiting from each fin of the hyperlens discretely. A metallic hyperlens is then custom-fabricated, and used to demonstrate for the first time, a super-resolved image with 5X magnification in the ultrasonic domain. The proposed hyperlens and the reception technique are among the first demonstrations in the ultrasonic domain, and well-suited for practical inspections. The results have important implications for higher resolution ultrasonic imaging in industrial and biomedical applications.

## Introduction

The resolution of imaging systems using classical waves is constrained by the diffraction limit, to half the operating wavelength^1^. This is largely due to the fact that evanescent scattered waves that carry information on fine features decay exponentially, dying out within the near field. Thus, imaging beyond the diffraction limit requires the successful extraction of information from the evanescent waves. One way to achieve this is by imaging within the near-field^2,3^. However, such approaches are significantly affected by poor signal-to-noise ratios achievable and hence the complexity in post-processing.

An alternative is to harness the information carried by the evanescent waves by successfully transferring them to far-field. This approach has attracted much interest in recent years with concepts such as negative indexed media, super-lenses and metamaterials in the electromagnetic^4–8^ and acoustic domains^9–11^. The authors have also demonstrated the application of periodic^12–14^ and non-periodic^15^ holey metamaterials for super-resolution imaging in the ultrasonic domain.

However, most metamaterial concepts require the acquisition of wavefields transmitted through their geometric features, which are of subwavelength order, to be useful in imaging applications^13^. Often this entails the use of sophisticated equipment such as Laser Doppler Vibrometers (LDV) to facilitate the acquisition of wavefields at fine spatial intervals. This challenges the practical implementation of metamaterial lenses, especially when they are miniaturized to achieve deeper subwavelength resolution. Hence, a mechanism to achieve super resolution along with magnification capability is of much practical interest. Such ‘super resolution with magnification’ concept was demonstrated in optical^16–20^ and acoustic^21–26^ domains with ‘hyperlenses’ that convert evanescent waves into propagating ones, thus carrying information to the far-field. Although the term ‘hyperlens’ was originally used for an ideal indefinite medium having hyperbolic dispersion^27^, it is also applicable for lenses with elliptical dispersion, as long as the dispersion is flat for a large range of wavevectors ^28^.

Among the various concepts proposed, the cylindrical hyperlens^22^ which has been demonstrated experimentally in the acoustic domain is relatively simpler in design and can operate over a broad range of frequencies since there is no local resonance involved. It consists of radially spreading metal fins which are arranged alternatively in the angular direction. The fins are of subwavelength dimensions with respect to the operating frequency and thus by using effective medium approaches, the dispersion of cylindrical hyperlenses can be written as^22^,

1 $$\frac{{k_{r}^{2} }}{{\rho_{r} }} + \frac{{k_{\theta }^{2} }}{{\rho_{\theta } }} = \frac{{\omega^{2} }}{B}$$

where $k_{r}$, $k_{\theta }$ and $\rho_{r}$, $\rho_{\theta }$ are the wavevectors and effective densities along the radial and angular directions respectively, *B* is the effective bulk modulus and $\omega$ is the operating frequency. The large difference between the densities of metal and air causes the effective density in the angular direction to be much higher than the radial one, which helps in achieving a flat dispersion enclosing high angular wavevectors. Also, the large ratio of the outer and inner radii compresses and converts the evanescent components into propagating waves when the waves propagate through the structure. The magnification factor of the hyperlens is typically determined by the ratio of the outer and inner radii.

Although several demonstrations of hyperlenses were reported in the electromagnetic and acoustic domains, their extension towards ultrasonic regime is rare^29,30^. Recently, the authors have studied the cylindrical hyperlens in the ultrasonic regime^31^. However, the use of hyperlenses for imaging defects in the context of ultrasonic nondestructive evaluation (NDE) and non-invasive diagnostics has not been reported. This is perhaps due to two pertinent challenges: firstly, the performance of the hyperlens in the ultrasonic regime has to be studied, in order to effectively design and fabricate it for practical experiments. Moreover, an effective approach for the reception of waves transmitted by the hyperlens is essential. Given the higher frequencies employed in ultrasonics (as compared to acoustics), the transmitted wavefield would need to be picked up carefully using sophisticated instrumentation, and doing this under water immersion is a significant further challenge.

In this article, we report work on the design and implementation of a hyperlens concept, along with an effective signal reception technique for subwavelength imaging of defects in the ultrasonic regime. Firstly, we gain confidence in our simulation approach by recreating experimental results from literature^22^ in the context of acoustic hyperlenses. We then make use of the numerical models to study the performance of cylindrical hyperlenses, gaining insight into their construction for the ultrasonic domain. A hyperlens is then fabricated, and used to demonstrate for the first time, a super-resolved image with 5X magnification in the ultrasonic domain, in new experiments at our Laboratory.

## Results

In order to demonstrate subwavelength imaging with magnification, we considered the problem of resolving two circular defects separated by a subwavelength distance—this scenario is often encountered in practical NDE. The defects considered were of subwavelength (λ/2.5) spacing with respect to the operating frequency of 250 kHz. A water immersion ultrasound imaging setup using ‘through-transmission’ configuration is considered. A cylindrical hyperlens, as reported in literature for the acoustic case^22^, was adopted. The hyperlens is composed of metallic fins with the ensuing channels filled with water during inspection. Finite Element (FE) simulations were used to further study the performance of the hyperlens and thus optimize its geometrical parameters as suited for practical realization. Firstly, to gain confidence in and verify our FE modeling approach, a cylindrical hyperlens, as reported in literature^22^, was recreated in our simulations. The close agreement of the FE results in comparison with the results reported in the literature (not shown here) verifies our FE modeling approach. 2D FE simulations using our thus-verified modeling approach were then carried out to study the effect of geometric and other parameters of the hyperlens on the performance of imaging defects in the ultrasonic (high frequency) domain under water immersion.

### Essential geometric parameters for the design of a cylindrical hyperlens

**Figure 1:**
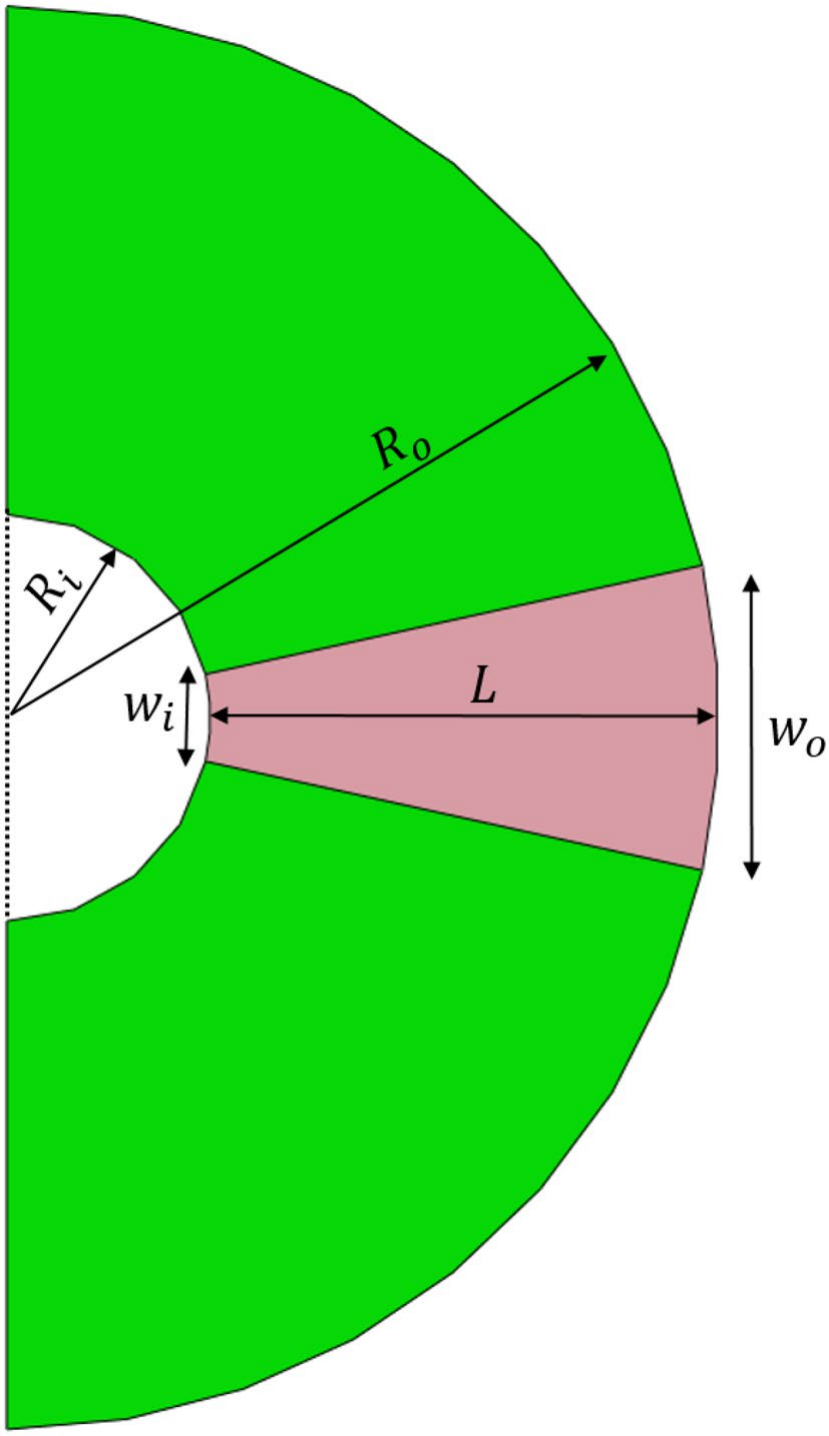
Schematic illustration of the geometric parameters involved in the design of cylindrical hyperlens. For the sake of brevity, a single fin is shown.

The parameters involved in the design of cylindrical hyperlens can be listed as *M, R*_i,_ *R*_o,_ *w*_i,_ *w*_o_ and *L.* Where *M* is the magnification factor, *R*_i_, *R*_o_ are the inner and outer radius of the semicircle forming the hyperlens*, w*_i_, *w*_o_ are the entry and exit width of the fins and *L* is the length of the fins. A schematic illustration of the hyperlens with model parameters is shown in Fig. 1. For the cylindrical hyperlens, the magnification factor *M* = *R*_o_/*R*_i_ (see Ref.^22^)_._ Based on this and the cylindrical geometry of the hyperlens, for a specific *M, R*_o_ and *w*_o_ can be determined as *M* times *R*_i_ and *w*_i_ respectively. Similarly, the length of the fin *L* can also be defined as (*M*−*1) R*_i_*.* Thus, the essential geometric parameters for designing a *M* times magnifying cylindrical hyperlens can be reduced to *R*_i_ and *w*_i_*.*

### Optimal inner radius of hyperlens

**Figure 2:**
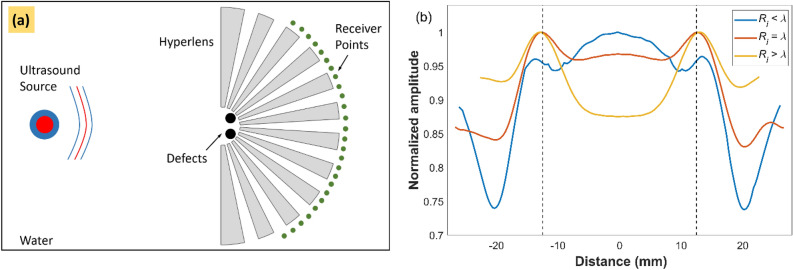
(**a**) Schematic illustrating the 2D FE model of the cylindrical hyperlens concept studied here for imaging defects in the ultrasonic domain. (**b**) Normalized pressure amplitudes obtained at the outer race of the hyperlens. Dotted lines indicate the expected location of the peaks in the magnified image.

Firstly, we studied the influence of the inner radius of the hyperlens on the imaging of subwavelength spaced defects. Three different cases were considered, *R*_*i*_ < λ, = λ and > λ, where *R*_*i*_ is the radius of the inner semicircle forming the hyperlens and λ is the wavelength at the operating frequency. The outer radius of the hyperlens *R*_*o*_ was considered as 10 times *R*_*i*_ in order to produce 10X magnification at the outer race. Figure 2a shows the schematic of the FE model considered where a Hanning windowed pulse, excited at 250 kHz, was used to image defects represented by the circular holes positioned in front of the hyperlens (more details on the FE model is given in "Methods" section below). Each of the fins in the hyperlens was modeled to have an entry and exit width of 100 µm and 1 mm which are subwavelength for the operating frequency (λ/60 and λ/6 respectively). The defects studied were of 1 mm diameter and separated by a center to center distance of 2.5 mm, which is subwavelength (λ/2.5) to the operating frequency. Figure 2b shows the envelopes of the obtained maximum signal amplitude outside the hyperlens for the cases studied. The formation of peaks corresponding to the defects is due to the scattering phenomena within the near-field, where the wavefields passing around the circular periphery of the defect meet again in the shadow region of the defect and interfere constructively. For the case of *R*_*i*_ < λ, the amplitude of defect peaks is much smaller and there is a high amplitude region in the middle which makes the peaks indistinguishable and prediction of defects challenging. Also, it can be observed from the results that while reducing *R*_*i*_, the amplitude of the defect peaks is reducing and the amplitude in the middle is increasing. The results show that the optimum performance of the hyperlens is obtained when the inner radius of the hyperlens *R*_*i*_ ≥ λ (detailed discussion of the results are given in "Discussion" section below).

### Optimal fin entry width

**Figure 3:**
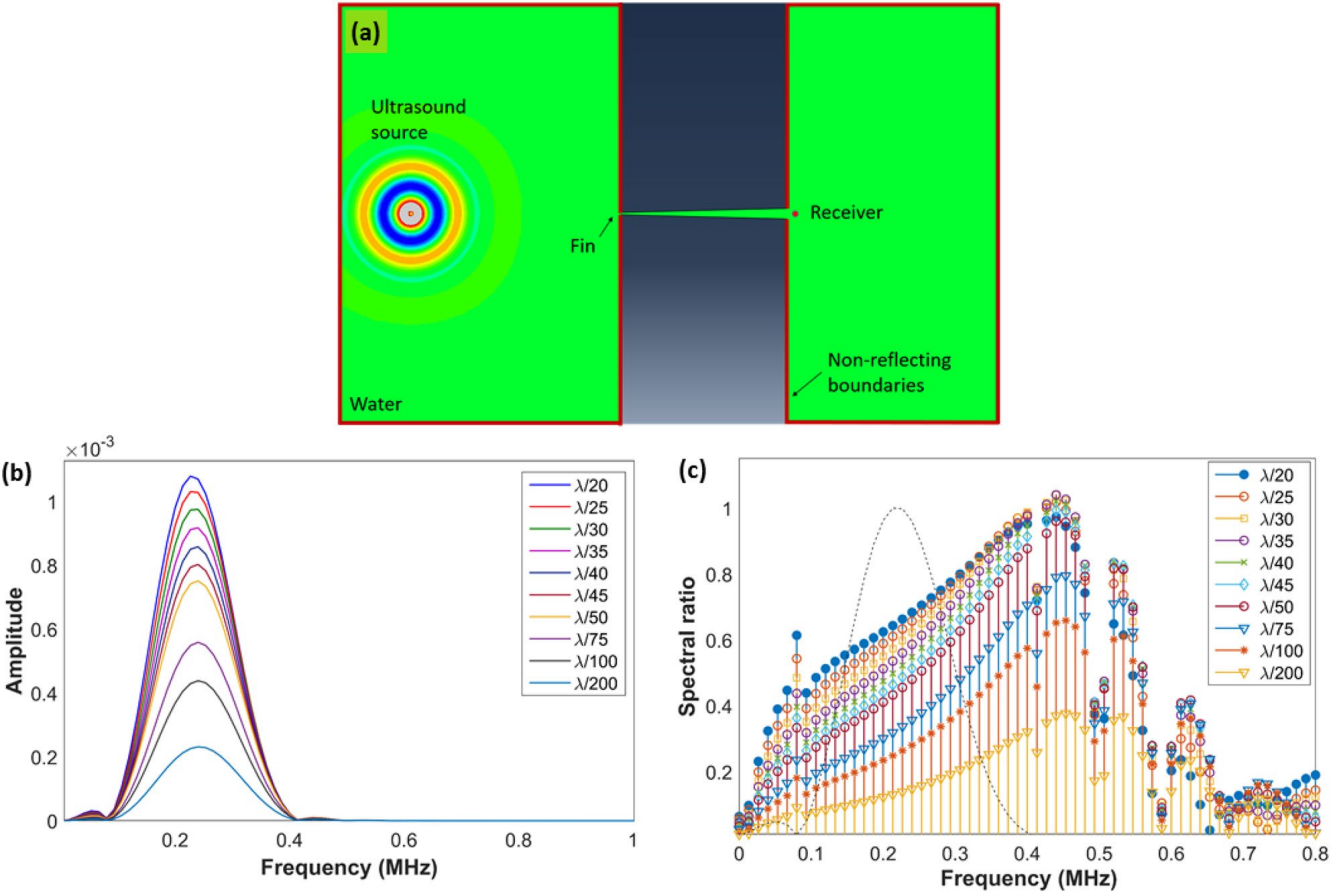
(**a**) Schematic of the FE model considered for the study of wave transmission past a single water-filled fin. (**b**) Frequency spectrum of the wave captured at the exit of the fin. (**c**) Spectral ratio obtained for various cases of *w*_*i*_. The normalized frequency spectrum of the wave received without hyperlens was shown as a dotted curve to indicate the signal's bandwidth.

Next, to optimize the entry width of the fins, a spectral analysis of the waves past the fins and captured at the exit of the hyperlens was performed. For this purpose, a single fin was modeled and various cases of entry width of the fin *w*_*i*_ were considered in terms of λ, and the exit width *w*_*o*_ was considered as 10*w*_*i*_ assuming a 10X magnifying hyperlens. Since the exit width must be ≤ λ/2 in order to be subwavelength, λ/20 is chosen as the upper limit for the entry width. The majority of the entry widths considered in the study are practically realizable using conventional machining techniques for low-frequency water immersion ultrasound imaging (for example, λ/60 = 0.5 mm for 50 kHz). The length of the fin, *L* was fixed as 9λ (i.e. *R*_*o*_*–R*_*i*_) by assuming the hyperlens inner radius *R*_*i*_ = λ. Spectral ratios for the different cases of *w*_*i*_ were calculated as the ratio of the frequency spectrum of the wave captured with fin and without fin, and the results are shown in Fig. 3. It can be observed from the results that the magnitude in the transmitted spectrum is directly proportional to *w*_*i*_ and there is no spectral loss associated with *w*_*i*_ while the fin length *L* is constant. The spectral ratio also increases with an increase in frequency for all cases of *w*_*i*_. From the results, it can be concluded that the fin entry width *w*_*i*_ does not significantly influence the transmission spectrum of the hyperlens besides the reduction in transmitted amplitudes.

### Subwavelength imaging of defects separated by λ/3

For further experimental demonstration of the above results in the ultrasonic domain, a metallic hyperlens was fabricated. The hyperlens was designed to have radially alternating fins composed of Stainless Steel (SS) and water (See "Methods" section below). Due to the complexities involved in the fabrication of the metallic hyperlens, the fins were designed to have an entry and exit width of 0.5 mm and 2.5 mm respectively, permitting a 5X magnification at 100 kHz. The operating frequency was thus set to 100 kHz to ensure that the geometrical parameters of the hyperlens are of subwavelength dimensions. A study on the effect of filling fraction on the dispersion characteristics of the metallic hyperlens was carried out and thus, a filling fraction of 0.5 was chosen (See "Discussion" section below). As scatterers (defects) for imaging, two metallic wires of 2.8 mm diameter were placed close to the inner race of the hyperlens with a center to center distance of 5 mm (λ/3).

**Figure 4:**
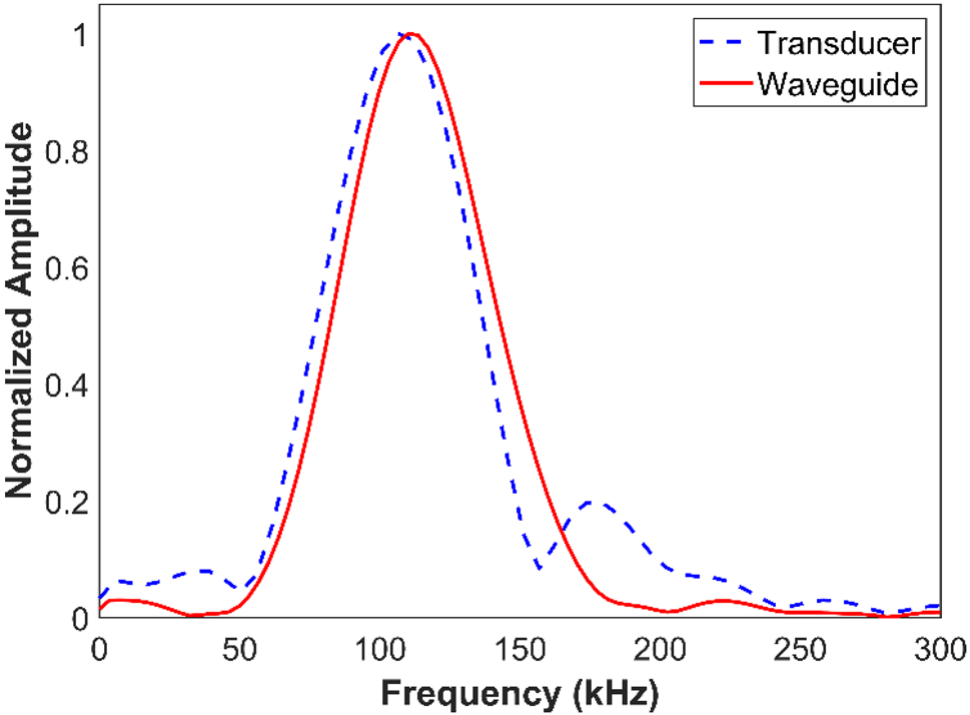
Experimental results showing the comparison of frequency spectrums received with and without waveguide attachment to the transducer. Waveguide allows broadband reception by not limiting the frequency range that the attached transducer can receive.

For successful imaging, the wavefields passing through each of the hyperlens fins must be captured discretely with fine spacing. Although the authors reported using Laser Doppler Vibrometer (LDV)^12–15^ and conical hollow horn^32^ for signal reception in previous studies, there are still challenges in the practical implementation of these approaches. In the work reported here, the authors implemented a novel waveguide-based reception technique that allows for a receiver with a finer aperture than the hyperlens’ features. Waveguides are structures with geometries finer than the operating wavelength and have been shown to be useful in various ultrasonic sensing applications^33–35^. Here, the authors' goal is to use a cylindrical waveguide to discretely capture the wavefields as transmitted by features with fine spacing. Also, the waveguides enable broadband reception as it does not employ any resonance phenomena which may induce losses in frequencies. Furthermore, a waveguide can be used for broadband operating frequencies, where only the velocity of the wave modes inside the waveguide change and it can be determined with the help of dispersion curves. In this work, a thin circular waveguide of 1 mm diameter was used as a receiver. Figure 4 shows the comparison of frequency spectrums received with and without waveguide and demonstrates the broadband reception of the waveguide receiver.

**Figure 5:**
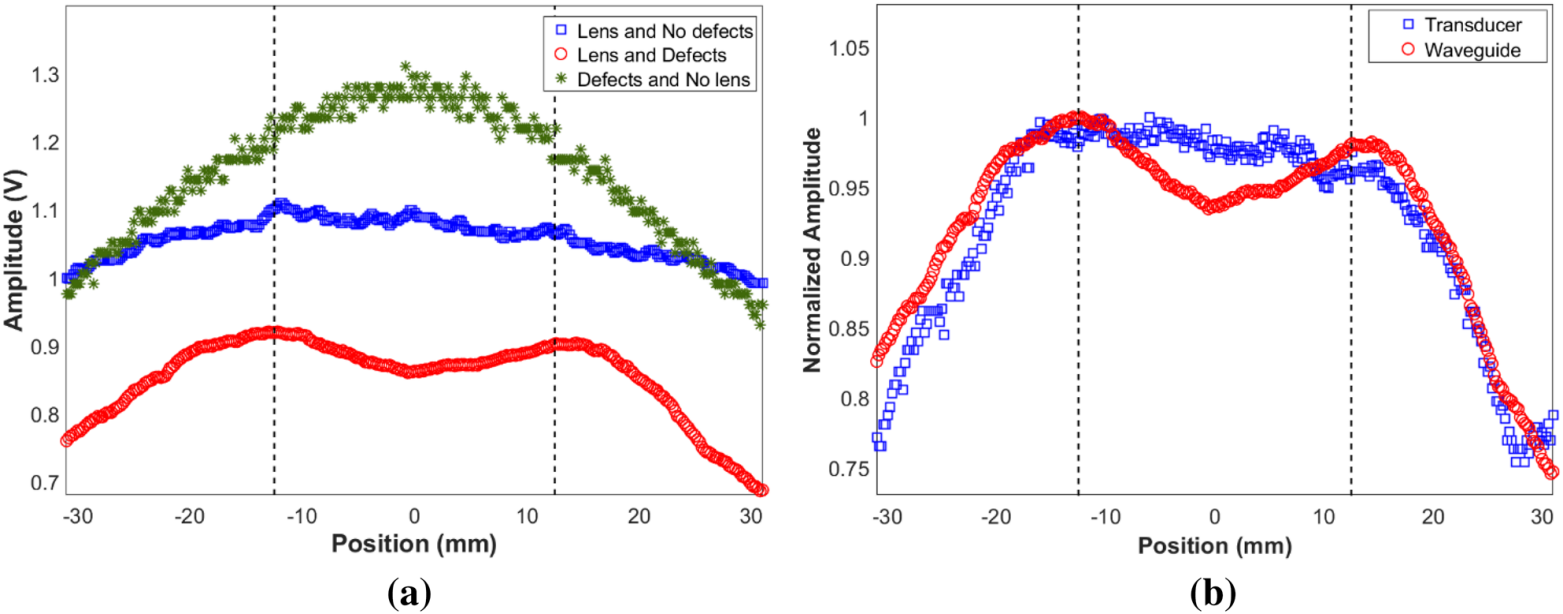
Experimental results from 5X hyperlens showing the magnified image for defects separated by a subwavelength distance of 5 mm (λ/3). (**a**) Results obtained using waveguide receiver. (**b**) Comparison of results obtained using waveguide receiver and commercial transducer. Since the amplitudes of signals received in both cases differ by nearly 20 dB for the same settings, the results are presented on a normalized scale. The dotted lines show the expected locations of the defects in the magnified image.

The waves that interact with the waveguide propagate as the lowest symmetric Lamb mode, with displacements in the axial direction that can be captured by placing a suitably oriented longitudinal or shear transducer. For ease of implementation, a shear transducer was considered, and a holder^33^ made of plexiglass was designed and fabricated for mounting on it the waveguide. Since waveguides can conduct ultrasound to longer distances, this process allows remote reception. However, in the studies reported here, the length of the waveguide was chosen so that the reverberations due to multiple reflections do not pollute the signal of interest. The signals were collected outside the rear edge of the hyperlens and the envelope of the maximum amplitudes was obtained (See "Methods" section for details on the experimental setup). Experimental results for the cases with and without the presence of the defects, are shown in Fig. 5a. The results show that the obtained peaks are well aligned with the expected locations of the scatterers in the 5X magnified image, experimentally demonstrating for the first time, a cylindrical hyperlens concept for imaging defects under water immersion in the ultrasonic regime. To demonstrate the effectiveness of the waveguide receiver in spatially narrowband reception, the results obtained with and without waveguide attachment to the transducer is shown in Fig. 5b. The typical active diameter of bulk ultrasonic transducers is much larger than the dimensions of fins of hyperlens. Thus, the transducer would capture scattered wavefields from the whole region of hyperlens corresponding to this area and the received signal is an average value of the wavefields from all the fins within that region. Hence, the information obtained from each fin in the hyperlens is not discretely captured and the defects are not perfectly resolved.

## Discussion

The results obtained for the studies on the geometrical parameters of the hyperlens can be discussed as below. The optimum performance of the hyperlens is obtained when the inner radius of the hyperlens *R*_*i*_ ≥ λ. This can be attributed to the elliptical dispersion characteristics and the cylindrical symmetry of the hyperlens that conserves angular momentum^16^. Since the angular momentum is conserved along the radial direction, the angular wavevector $k_{\theta }$ increases towards the origin and exponentially decays away elsewhere. However, due to the elliptical dispersion characteristic of the hyperlens, at any given point, the radial wavevector $k_{r}$ tends to zero with the increase in angular wavevector $k_{\theta }$, which sets a lower limit for the inner radius *R*_*i*_. This lower limit of *R*_*i*_ can be found by setting $k_{r}$ to zero in the dispersion relation presented in Eq. (1). For an isotropic medium following a circular dispersion, the lower limit of *R*_*i*_ is ⁓ λ. On the other hand, for an anisotropic medium having a truly hyperbolic dispersion, the lower limit of *R*_*i*_ is much smaller than λ as $k_{r}$ is not vanishing but increasing with increase in $k_{\theta }$. For the cylindrical hyperlens with an elliptical dispersion, the lower limit of *R*_*i*_ is smaller than λ due to the larger major axis. This lower limit *R*_*i*_ is mainly controlled by the material properties of the fins considered. In general, the inner radius can be considered comparable to λ as it satisfies the lower limit for all the dispersion relations discussed above.

On the other hand, the fin entry width does not significantly influence the performance of the hyperlens as shown by the spectral ratios in Fig. 3. It is worth noting here that for all the cases considered here, the waves propagating inside the fin are of the fundamental plane wave mode and other higher order modes do not exist since the size of the fin is much subwavelength^36^. The observed decrease in the magnitude of the spectrum with reduction in the entry width is due to the fact that the energy of the wave propagating inside the fin is directly proportional to the size of the fin^36^. Besides the amplitude of the signal, the fin width can also alter the maximum resonance frequency^37^, however that is not significant here as the working principle of the cylindrical hyperlens does not involve any resonance. Thus, it can be concluded that the fin entry width is not significantly altering the performance of the hyperlens, however, for the effective medium description to be valid, the fin entry and exit width dimensions must be subwavelength as compared to the operating frequency^22^.

**Figure 6:**
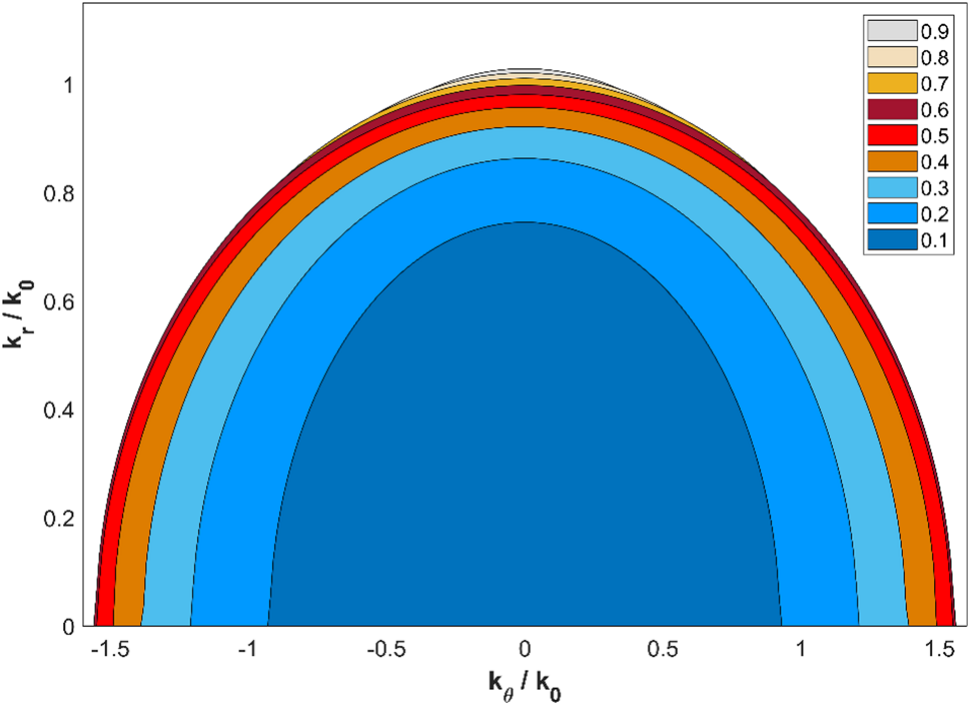
Dispersion curves of the cylindrical hyperlens composed of water and stainless steel fins with various filling fractions.

**Figure 7:**
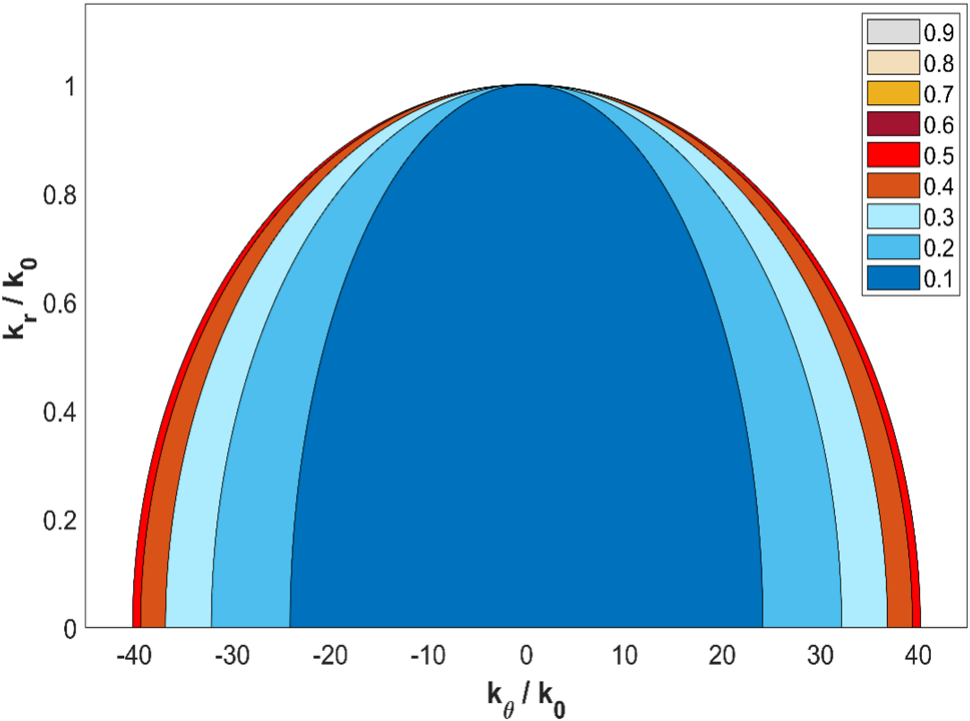
Dispersion curves for the cylindrical hyperlens composed of air and stainless steel fins with various filling fractions.

In addition to the geometrical parameters of the fins, the filling fraction of the fins is another factor to be studied for the optimal performance of the hyperlens. Unlike the fin entry width and inner radius of the hyperlens, the filling fraction is a material related parameter and its influence on the performance of the hyperlens can be studied using the dispersion curves as discussed here. For the materials considered here, the optimum filling fraction to enable higher angular wavevector $k_{\theta }$ which typically carries the evanescent wave components was studied using the dispersion characteristics of the hyperlens given in Eq. (1). Using effective material properties^22^, the elliptical dispersion curve of the hyperlens made of water and Stainless Steel (SS) was computed for various filling fractions of the fin and the results are shown in Fig. 6. The dispersion curve with the largest $k_{\theta }$ was obtained for a filling fraction of 0.6. However, in the present work, a filling fraction of 0.5 was considered as set by the limits of the fabrication process in machining the minimum fin entry width *w*_i_. Further to emphasize the fact that the filling fraction is a material dependent parameter, for a comparison purpose, dispersion curves were also computed for the hyperlens composed of air and SS fins and shown in Fig. 7. It can be seen from the Fig. 7 that the dispersion curves are more flatter and cover larger angular wavevector $k_{\theta }$ than the water filled hyperlens. This is because of the higher density difference achieved between air and SS than water and SS material. Also we note that for this case, the largest $k_{\theta }$ was obtained for a filling fraction of 0.5.

**Figure 8:**
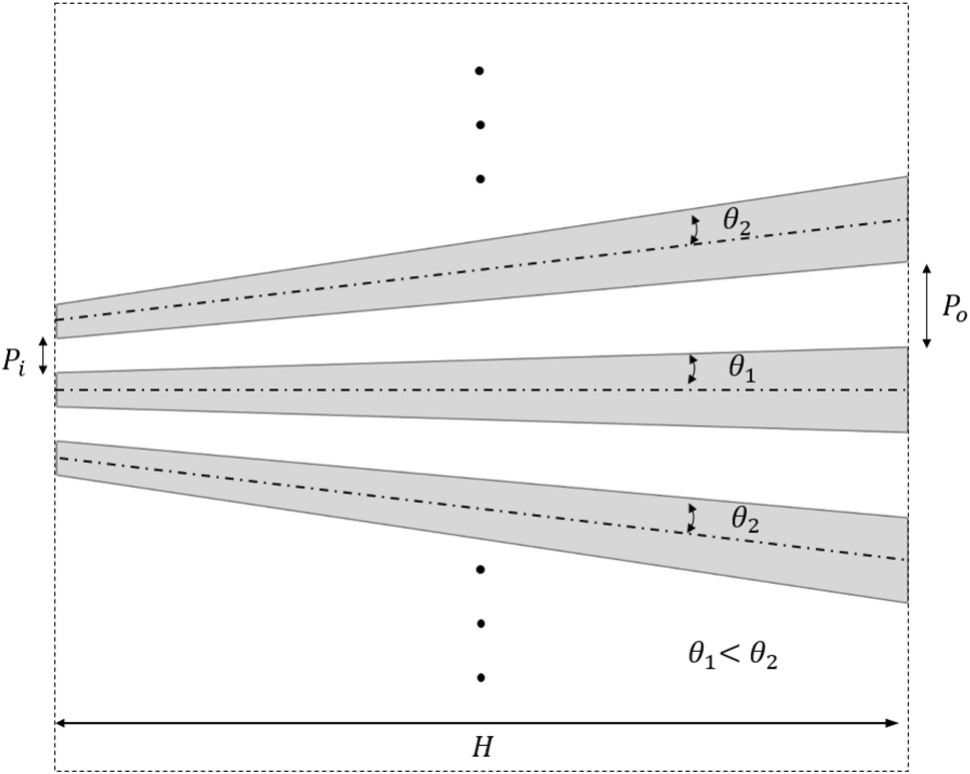
Schematic illustration of the design of constant period flat face hyperlens.

Although the cylindrical hyperlens demonstrates the super resolving capability, the curved form of the lens limits its practical implementation since the objects need to be placed inside the inner semi-circle of the lens. Hence, an extension of the concept from cylindrical to planar conformation was proposed to have flat/ planar input/output faces. The design of the flat hyperlens was adopted from the design of cylindrical hyperlens by assuming both the inner and outer radii *R*_*i*_ and *R*_*o*_ are >  > λ . Hence, the periodicities of the fins (*P*_*i*_ and *P*_*o*_) at the inner and outer flat faces of the hyperlens were also considered as constant. As per the assumptions made here, the angle of the fin (ѳ) will be constant for all fins of the lens. However, for a finite width of the lens, *H*, and fixed periodicities on both faces, the angle (ѳ) will vary from fin to fin across the lens and thus the length of the fins also varies. Figure 8 shows the schematic illustration of the design of flat face hyperlens.

**Figure 9:**
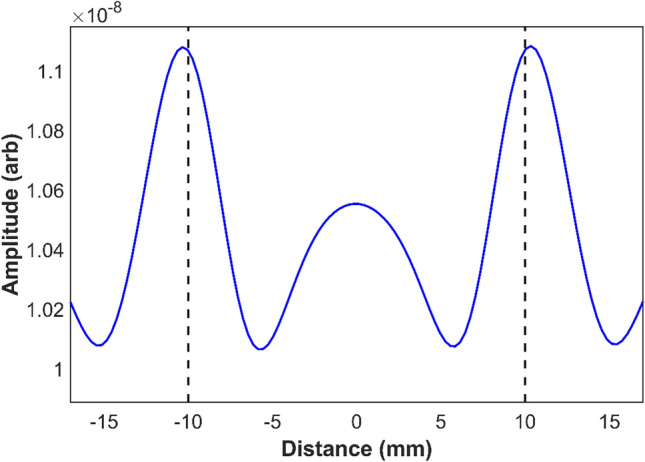
Numerical results from 10X magnifying flat face hyperlens showing the magnified image for defects separated by a subwavelength distance of 2 mm (λ/3). The dotted lines show the expected locations of the defects in the magnified image.

To demonstrate the flat face hyperlens, a numerical model was developed by adopting the modelling configuration followed in the studies described above for optimum inner radius of cylindrical hyperlens. The operating frequency was considered as 250 kHz. The width of the hyperlens was chosen as 48 mm. The lens was designed for a 10X magnification and each of the fins in the hyperlens was modeled to have an entry and exit width of 100 µm and 1 mm respectively. The periodicities of the fins at inner and outer faces also were considered as 100 µm and 1 mm respectively. The defects studied were of 1 mm diameter and separated by a center to center distance of 2 mm, which is subwavelength (λ/3) to the operating frequency. The results obtained from the numerical simulation is shown in Fig. 9. The results show very good agreement with the expected 10X magnified super resolved image of the defects. However, there is a high amplitude region at the middle, which is due to the shorter lengths of fins in the center region of the hyperlens. Moreover, because of the unequal fin lengths, there is time delay between the wavefields emitting from each fin. Hence, the time delay must be accounted while processing the results. Nevertheless, the flat face hyperlens is well-suited for practical inspections and has important implications for higher resolution ultrasonic imaging in industrial and biomedical applications. Improvisation of the design for more effectiveness is in progress.

## Conclusion

This paper presented the development and application of a hyperlens for ultrasonic regime that can magnify subwavelength features and achieve super-resolution in the far-field. Numerical simulations were used to study the performance of hyperlenses with regard to their geometrical parameters in imaging defects separated by a subwavelength distance. An elegant extension of the concept of cylindrical hyperlens was proposed to have flat faces for wider practical implementation. The paper also demonstrated a novel waveguide-based reception technique that uses a conventional ultrasonic transducer as receiver to capture waves exiting from each fin of the hyperlens discretely. A practical super resolution imaging of defects separated by one-third of the operating wavelength was demonstrated for the first time with 5X magnification in the ultrasonic domain. The proposed hyperlens and the reception technique are among the first demonstrations in the ultrasonic domain, and well-suited for practical inspections. The results have important implications for higher resolution ultrasonic imaging in industrial and biomedical applications.

## Methods

### FE model

A commercial FE software package^38^ was used to simulate the wave propagation in the hyperlens. 2D plane strain conditions were assumed and linear quadrilateral acoustic elements were used for the model. The numerical model was designed to simulate immersion ultrasound configuration and we assumed the hyperlens to be composed of alternating layers of water and metal fins. The model was assigned with water properties. The metallic fins were realized by setting rigid boundary conditions on the respective fin boundaries. The defects were modeled as circular holes. A Hannnig pulse excitation was given to a node positioned at a sufficient distance from the defects and hyperlens to ensure plane wave incidence. The outer boundaries of the model were assigned with non-reflective boundary conditions to avoid unwanted reflections. The mesh size and the incremental time step were chosen such that they satisfy the established convergence and stability criteria. (For more details on the FE modeling, see Ref.^31^ and the references therein).

### Experiments

**Figure 10:**
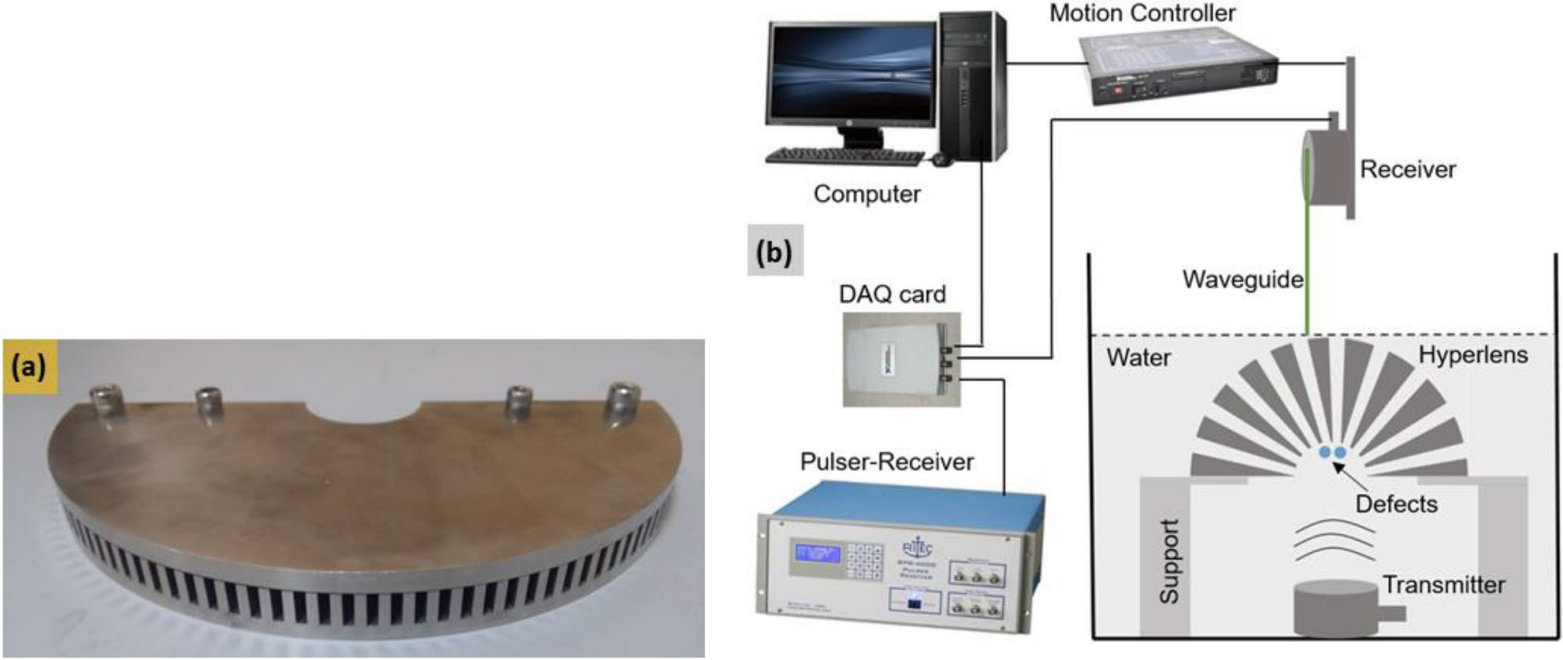
(**a**) Photograph of the metallic hyperlens used in experiments and (**b**) Schematic illustration of the experimental setup.

The metallic hyperlens was fabricated by machining the fins on a 12.5 mm thick SS plate using Electric Discharge Machining (EDM) for a depth of 10 mm resulting in a substrate of 2.5 mm in thickness. The top side of the hyperlens was covered by another 2.5 mm thick SS plate to confine the wave propagation within the fins. Figure 10a presents a photograph of the fabricated metallic hyperlens. The experiments were carried out in a water immersion setup using through-transmission configuration. A commercial transducer with a center frequency of 100 kHz (05L007, Valpey Fisher) was employed as a transmitter. A 3 cycle Hanning-windowed toneburst centered at 100 kHz was generated by a RITEC 4000 Pulser-Receiver (Ritec Inc., USA) and it was applied to the transmitter to excite the wave propagation in water. The scatterers were placed near the inner race of the hyperlens such that the scattered wavefield from the scatterers can be coupled into the hyperlens. The transducer holder, which mounted the receiving transducer and the waveguide receiver, was attached to a raster scanning system and used to capture the scattered wavefield on the output side of the hyperlens. Figure 10b illustrates the experimental setup.

## Data Availability

The data that support the findings of this study are available from the corresponding author upon reasonable request.
